# Growth, Protein and Energy Intake in Children with PKU Taking a Weaning Protein Substitute in the First Two Years of Life: A Case-Control Study

**DOI:** 10.3390/nu11030552

**Published:** 2019-03-05

**Authors:** Sharon Evans, Anne Daly, Jo Wildgoose, Barbara Cochrane, Satnam Chahal, Catherine Ashmore, Nik Loveridge, Anita MacDonald

**Affiliations:** 1 Birmingham Women’s and Children’s Hospital NHS Foundation Trust, Birmingham B4 6NH, UK; a.daly3@nhs.net (A.D.); satnamc@hotmail.com (S.C.); catherine.ashmore@nhs.net (C.A.); anita.macdonald@nhs.net (A.M.); 2 Bradford Teaching Hospitals NHS Trust, Bradford BD9 6RJ, UK; jo.wildgoose@bthft.nhs.uk; 3 Royal Hospital for Children Glasgow G51 4TF, UK; barbara.cochrane@ggc.scot.nhs.uk; 4 Danone Early Life Nutrition, Macquarie Park, New South Wales, Australia; nik.loveridge@danone.com

**Keywords:** Phenylketonuria (PKU), growth, protein substitute, weaning

## Abstract

Growth issues have been observed in young children with phenylketonuria (PKU), but studies are conflicting. In infancy, there is an increasing trend to introduce a second-stage semi-solid weaning protein substitute (WPS) but there is concern that this may not meet energy requirements. In this longitudinal, prospective study, 20 children with PKU transitioning to a WPS, and 20 non-PKU controls were observed monthly from weaning commencement (4–6 months) to 12 m and at 15, 18 and 24 months of age for: weight, length, head circumference, body mass index (BMI), energy and macronutrient intake. Growth parameters were within normal range at all ages in both groups with no significant difference in mean z-scores except for accelerated length in the PKU group. No child with PKU had z-scores < −2 for any growth parameter at age 2 years. Total protein and energy intake in both groups were similar at all ages; however, from 12–24 months in the PKU group, the percentage of energy intake from carbohydrate increased (60%) but from fat decreased (25%) and inversely for controls (48% and 36%). In PKU, use of low volume WPS meets Phe-free protein requirements, facilitates transition to solid foods and supports normal growth. Further longitudinal study of growth, body composition and energy/nutrient intakes in early childhood are required to identify any changing trends.

## 1. Introduction 

Phenylketonuria (PKU) is an inherited metabolic disorder caused by a deficiency of the enzyme phenylalanine hydroxylase resulting in neurotoxic accumulation of the amino acid phenylalanine (Phe). The central focus of treatment is dietary management from diagnosis (<10 days of age), comprising a very low natural protein (low phenylalanine) diet (commonly <10 g/day) supplemented with a synthetic Phe-free amino acid substitute formula to meet the energy and protein requirements for growth and to maintain acceptable blood Phe control [1,2]. 

Since the 1950s, although low Phe dietary treatment has been known to prevent severe neurological impairment [3], several studies have suggested that early growth may be suboptimal in young children [4,5,6,7,8,9,10,11] compared with reference populations, particularly with respect to reduced height and head circumference. It is postulated that this might be attributed to a lack of total protein, natural protein and/or energy intake. Other studies have found little or no difference in growth [12,13,14,15,16,17]. However, disparities in study findings might be attributed to geographical area, small subject numbers, retrospective or uncontrolled data and changes in dietary practices over time.

As with all children, introduction of solid foods (weaning) is recommended from 17 to 26 weeks of age [18,19]. However, solid foods can suppress the appetite for liquids, potentially lowering intake of infant protein substitute, thereby affecting the ability to achieve total protein requirements. As a consequence, it often becomes necessary to introduce a second stage, more concentrated protein substitute [20] at around 6 months of age. However, there is concern that the low energy density of weaning protein substitute (8 kcal/g protein) might not compensate for the energy content of infant protein substitute (33.5 kcal/g protein) and this may impact on growth and weight gain. Whilst evidence is limited, previous retrospective data has demonstrated that a weaning protein substitute can be introduced without adverse effects on appetite or growth [21]. However, no studies have prospectively studied growth, protein and energy intake of children during the weaning period and early years. 

Weaning in PKU is particularly complex and challenging as parents try to coordinate the normal transition from liquid formula to solid foods with the additional challenges of introducing a second stage protein substitute. They also must introduce special low protein foods in addition to the natural protein containing foods that will eventually replace normal infant formula or breast milk. Feeding problems are known to be more common in young children with PKU compared to non-PKU controls [22] and so the weaning period may be a particularly vulnerable period for optimal growth attainment.

## 2. Materials and Methods

This was an open label, longitudinal prospective case-control study in 20 children with PKU and 20 healthy controls. Subjects were recruited to assess their growth, energy and macronutrient (protein, fat and carbohydrate) intake from the introduction of a second stage, low volume, Phe-free protein substitute (3–6 months of age) to the age of 2 years (total of 17–20 months follow-up).

### 2.1. Anthropometry

Body weight, length, head circumference and Body Mass Index (BMI) were measured and recorded and z-scores calculated at each monthly review (from weaning until 12 months), then at 15, 18 and 24 months of age for all subjects. Weight was measured to the nearest 10 g using Seca (Seca, Hamburg, Germany) electronic infant scales; length was recorded to the nearest 1 mm using a Seca mobile measuring mat; and head circumference was measured to the nearest 1mm using a flexible, non-stretch head circumference tape. Self-reported paternal and maternal heights were also recorded for both groups. Length-for-age-difference, the difference between actual length (cm) and WHO standard length (cm) for that age at the 50th percentile [23], was also calculated as a more appropriate measure of change in linear growth over time than z-scores as they do not rely on standard deviations from cross-sectional data.

### 2.2. Dietary Records

A 24 h dietary record (including intake of weaning protein substitute, infant protein substitute and prescribed natural protein intake for PKU subjects) was completed by parents/carers monthly from weaning commencement to 12 months of age and then at 15, 18 and 24 months of age for all subjects. These were then cross checked by the research dietitian. Nutrient intake from diet records were assessed using the computer software nutritional analysis program Nutritics (Nutritics Professional Premium v5.09, Libro v0.9, Dublin, 2018) (food composition data from: 2015 COFIDS including McCance and Widdowson 7th edition, 2015).

### 2.3. Study Product (PKU Subjects)

The weaning protein substitute (PKU Anamix First Spoon; Nutricia Ltd, Trowbridge, UK) was a powdered Phe-free protein substitute supplemented with long chain polyunsaturated fatty acids (LCP’s), containing essential and non-essential amino acids, carbohydrate, fat, vitamins, minerals and trace elements. The product was mixed with water to produce a semi-solid spoonable protein substitute and 5 g of protein substitute powder provided 2 g of protein and 16 kcal. 

### 2.4. Introduction of Weaning Protein Substitute (PKU Subjects)

The weaning protein substitute was gradually introduced into the diet after low protein solids were established. This was at the same time that the Phe in breastmilk or standard infant formula was replaced with Phe from food sources. Subjects continued to drink Phe-free infant formula as their main source of Phe-free L-amino acids during the early stages of weaning. The clinical dietitian determined the dose and increments of the weaning protein substitute.

Data on qualitative aspects of feeding development, gastro-intestinal symptoms, the feeding environment, feeding practices and progression (texture of foods, self-feeding, where meals are consumed), and weaning problems (e.g., teething/illness) are reported in a separate publication.

### 2.5. Ethical Approval

This study was conducted according to the guidelines laid down in the Declaration of Helsinki and favourable opinion was given by the local research ethics committee (West Midlands–South Birmingham). Written informed consent was obtained from parents/carers of all children. 

### 2.6. Statistics

As an observational study, no formal hypothesis comparing the two treatment groups and no formal power calculation was used to determine the sample size. The number of patients chosen was a pragmatic choice allowing for reliable estimation of continuous covariates and deliverable within a reasonable timeframe based on the average number of subjects diagnosed with PKU per year (approximately 4–6 per centre).

Quantitative outcome measures were summarised, and descriptive statistics of the data presented. The growth of infants was compared with standard growth curves for healthy infants [23]. The change in subject measures over time were analysed using longitudinal modelling techniques including time as a continuous covariate and allowing for random intercept and slopes. Models were adjusted for gender with gender-by-time and treatment-by-time interactions to assess different trends between subject groups. Between-group differences for growth, energy, protein, carbohydrate and fat intake at defined time-points were analysed using unpaired t-tests. Statistics were analysed using R version 3 computer software.

## 3. Results

### 3.1. Subjects

#### 3.1.1. PKU Group

Twenty children (14 male) with PKU diagnosed by newborn screening, commencing weaning and requiring a concentrated phenylalanine-free protein substitute were recruited from three specialist PKU centres prospectively (Birmingham Children’s Hospital (*n* = 17), Bradford Teaching Hospitals NHS Trust (*n* = 2), and the Royal Hospital for Children, Glasgow (*n* = 1)). Subjects were recruited and reviewed by the same dietitian at each centre. 

#### 3.1.2. Control Group

Twenty control children (12 male; 18 matched with the PKU group for mother’s educational level and 18 matched for birth order) also commencing weaning were recruited from the local community. They included siblings of inherited metabolic disorder (IMD) patients, or children recruited from a local community centre. The caregivers of control subjects received no dietary advice from one of two research dietitians. 

All subjects were white Caucasian except for two control children of mixed 50% Caucasian, 50% Afro-Caribbean decent. Five children from the PKU group and one from the control group had a sibling with PKU and three carers in both groups were single parents. 

Median age for diagnosis of PKU was 10 days (range: 2–16 days). Median age of weaning was 4.3 m (range: 2.9–6.6 m) for PKU and 5.1 m (range: 3.7–6.5 m) for controls (*p* = 0.04). The decision to commence weaning (introduction of solid foods) was initiated by parents/caregivers in both groups. For children with PKU, the prescribed target total protein intake was 3 g/kg/day (2) and median natural protein intake at weaning was 5 g/day (range: 3–7 g/day). Two children were born prematurely at 33 weeks; corrected age (calculated by subtracting the number of weeks of prematurity from the chronological age) was used for calculating anthropometry.

### 3.2. Growth

**Figure 1:**
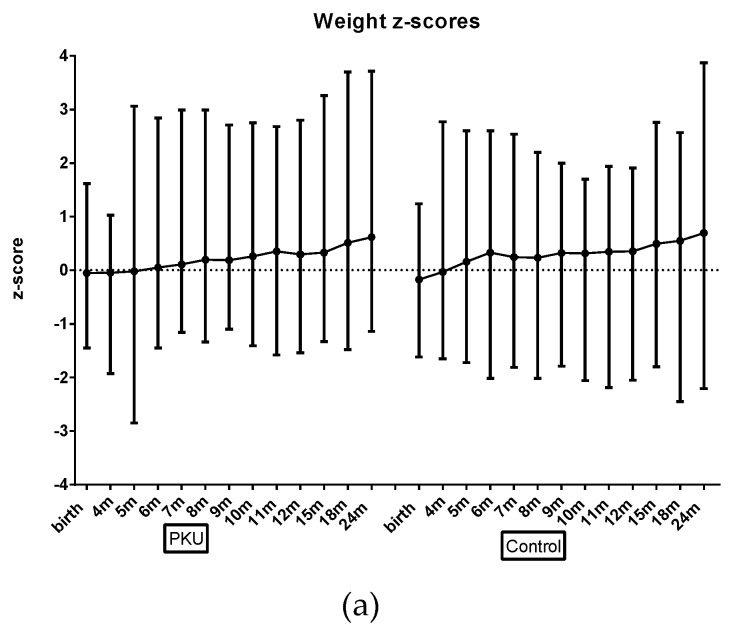

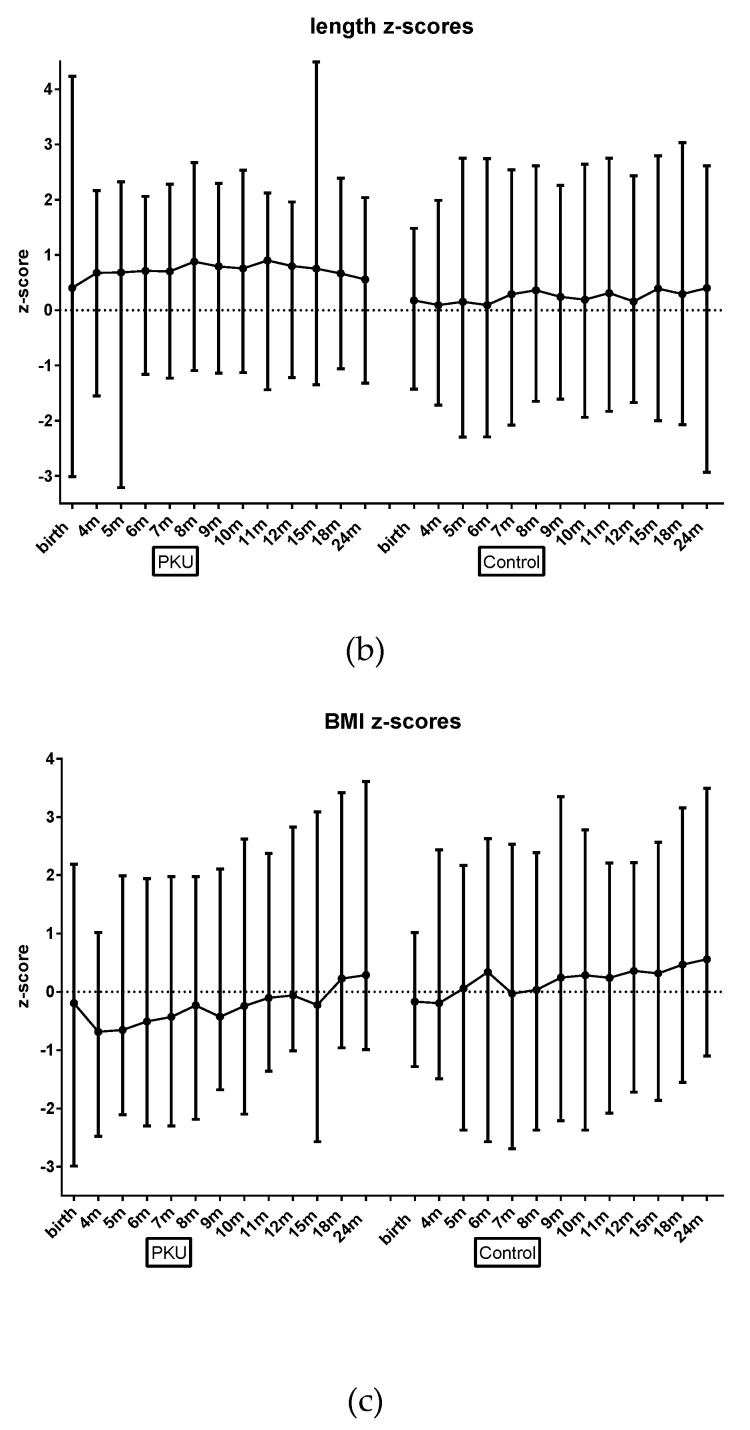
Mean weight, length and body mass index (BMI) z-scores for PKU and control groups by age. (**a**) Mean weight z-scores for PKU and control groups by age; (**b**) Mean length z-scores for PKU and control groups by age; (**c**) Mean BMI z-scores for PKU and control groups by age.

Longitudinal modelling showed that there was no significant difference between the PKU and control group for mean z-scores for (weight, head circumference or BMI) with both groups within normal range at all ages (Figure 1; Tables S1–S3). There was a trend toward slightly larger z-score length measurements (average increase of 0.73; *p* = 0.003; Table S4) for children with PKU; and consequently, slightly lower BMI, although not significantly (*p* = 0.08). Similarly, when length-for-age-difference was analysed using longitudinal modelling, children with PKU demonstrated significantly larger differences (mean difference between groups of 1.0 cm (0.7–1.6); *p* = 0.02; Table S5). No children with PKU had weight, length or BMI z-scores below −2 at 12 months or 24 months of age and only one control child had a weight and length z-score below −2 at 12 m and 24 months of age. Conversely, two control children and 1 child with PKU were overweight (BMI z-score > 2) at 12 months and 24 months. 

**Figure 2:**
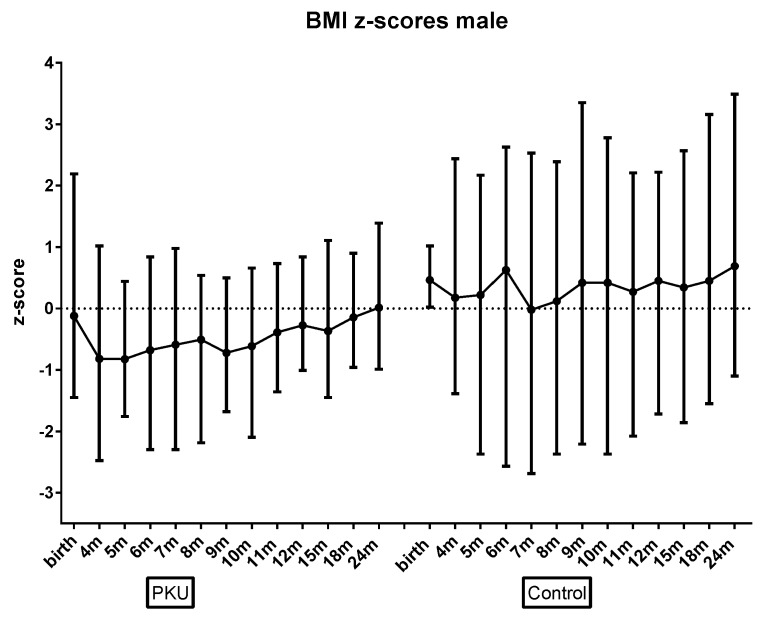
Mean **male** BMI z-scores for PKU vs. control groups by age.

**Figure 3:**
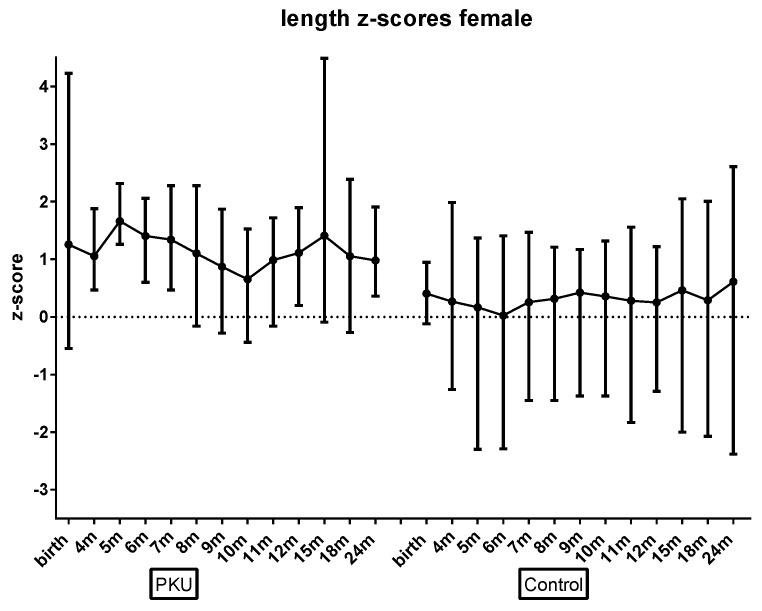
Mean female length z-scores for PKU vs. control groups by age.

**Figure 4:**
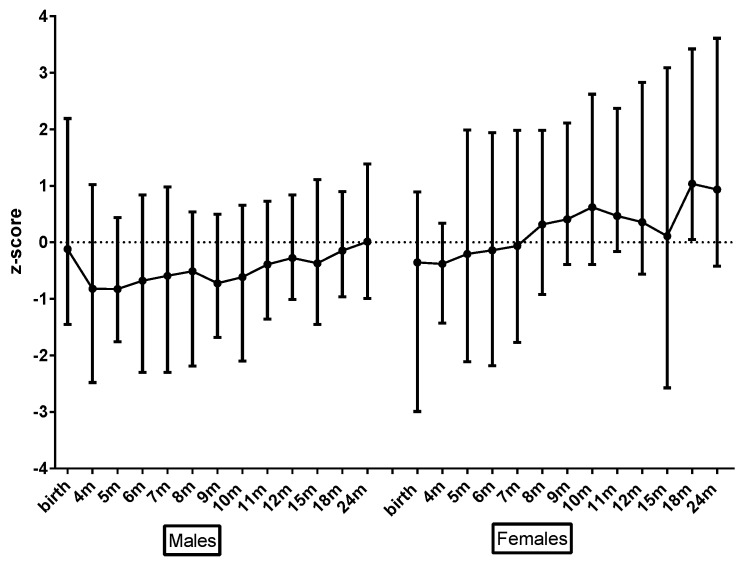
Mean PKU BMI z-scores for male vs. female by age.

There were some gender differences. Boys had lower mean BMI z-scores than girls across both groups (*p* = 0.006). In addition, whilst not statistically significant, there was a trend for boys with PKU toward lower BMI z-scores than controls (Figure 2); girls with PKU had length z-scores higher than control girls (Figure 3); and girls with PKU had higher weight, and subsequently higher BMI z-scores (Figure 4) than boys with PKU. Differences in length z-scores were not associated with genetic height potential as parental heights did not differ significantly between PKU and control groups.

### 3.3. Protein Intake from Protein Substitutes (PKU Group Only) (Excluding Natural Protein Intake)

**Figure 5:**
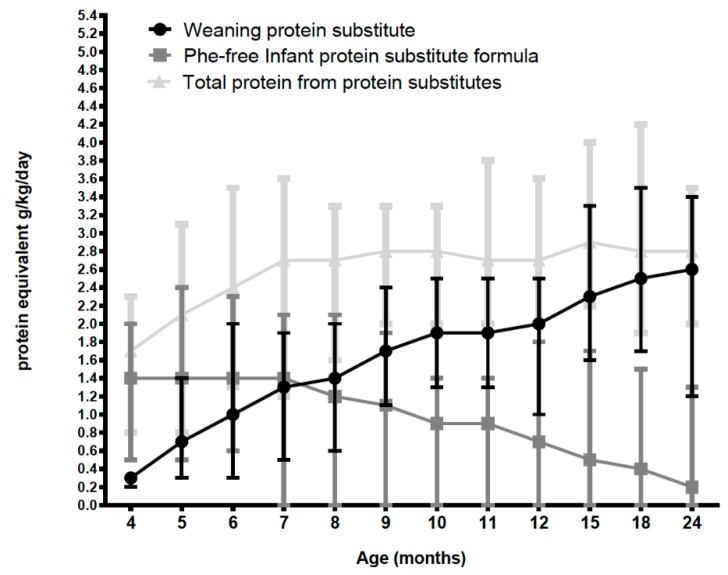
Mean protein intake g/kg/day from total protein substitute, weaning protein substitute, and Phe-free infant formula.

As protein intake from weaning protein substitute increased in an almost linear fashion, Phe-free infant formula intake decreased, but mean total protein intake from protein substitutes remained relatively constant at around 2.8 g/kg/day (2.7–2.9 g/kg/day) from 7 months of age (Figure 5). Phe-free infant formula intake continued to make a significant contribution to protein intake (mean of 500–600 mL/day) until 7 months of age, then gradually declined so that by 24 months of age, 75% (*n* = 15) of subjects had stopped Phe-free infant formula and the remaining five subjects had a mean of 407 mL/day (200–820 mL). By 12 months of age weaning protein substitute contributed a total protein intake of 60% and by 24 months 74%. Natural protein intake remained relatively constant at all ages at a mean of 0.7–0.8 g/kg/day and 20–23% of total protein intake from 6 months of age. 

### 3.4. Total Protein Intake PKU vs. Control (Includes Natural Protein Intake)

At all ages, children in both groups exceeded 100% of WHO/FAO/UNU safe protein intakes [24] and the safe-levels plus 40% (mean PKU: 194%, range: 141–251%; mean control 188%, range: 133–272%) recommended for PKU (1). Average total protein intake (g/kg/day) increased slowly over time (mean 0.07; *p* < 0.0001) although this increase was greater in the control group (*p* = 0.02; Table S6). Whilst there was a trend towards higher total protein intakes in the PKU group compared to the controls aged up to 10 months, the reverse was true from 11 months of age. Mean protein intakes ranged from 2.5–3.6 g/kg/day for children with PKU and 2.4–4.0 g/kg/day for controls with both groups achieving ≥ 3.0 g/kg/day from 6 months of age in the PKU group, and from 7 months in the control group.

When protein was expressed as a ratio of energy intake (g protein/100 kcal), from 6 months of age children with PKU consumed a consistent 3.4–3.7 g/100 kcal whilst controls had a larger range of 2.7–4.2 g/100 kcal (*p* = 0.001; Table S7). 

### 3.5. Total Carbohydrate and Fat Intake PKU vs. Control

**Figure 6:**
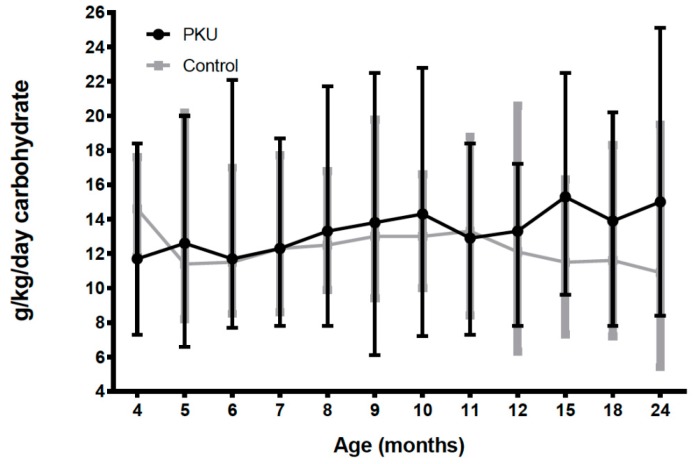
Mean carbohydrate intake (g/kg/day) for PKU and control groups by age.

Until 12 months of age, carbohydrate and fat intake was similar in both groups. However, from 1–2 years of age, children with PKU had significantly higher intakes of carbohydrate than controls and longitudinal modelling showed a statistically significant difference in the change in carbohydrate intake over time between PKU and control groups (*p* < 0.01; Table S8) (Figure 6). Conversely, after 12 months of age, fat intake was higher for control children although this only reached significance at 15 months ((g/day) 36 vs. 44, *p* = 0.02).

### 3.6. Energy Intake from Protein Substitutes (PKU Group Only) (Excludes Energy Contribution from Food)

**Figure 7:**
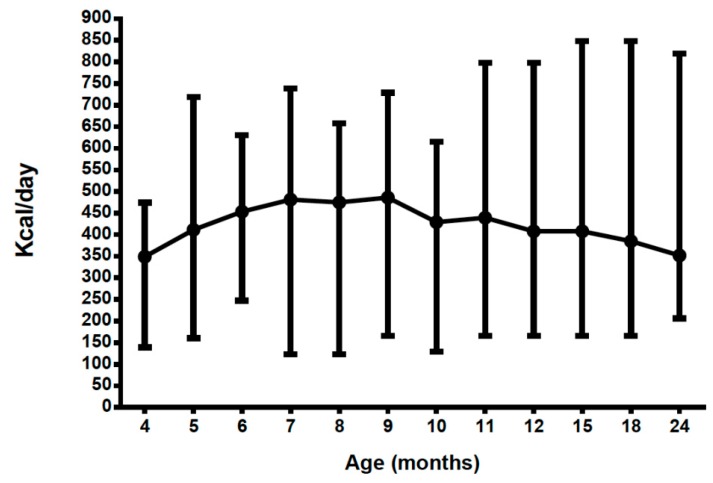
Mean energy intake (kcal) from both protein substitutes (infant and weaning) by age.

Energy intake from prescribed protein substitutes remained relatively constant at a mean of 423 kcal/day (349–486 kcal/day) (Table 1, Figure 7). 

### 3.7. Total Energy Intake PKU vs. Control (Includes Energy Intake from Food)

**Figure 8:**
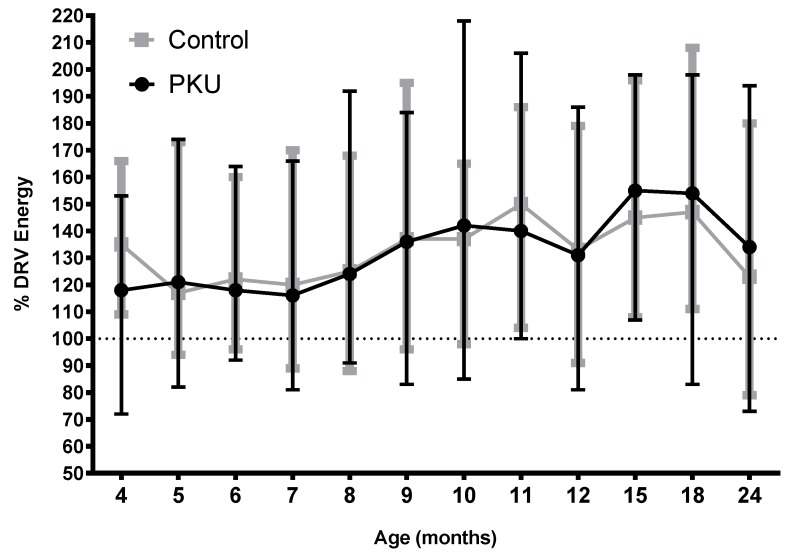
Mean % dietary reference values (DRV) for Energy for PKU and control groups by age.

There was no significant difference between PKU and control groups for total energy intake or total energy intake as a percentage of dietary reference values (DRV) [25] longitudinally or at any age (Figure 8; Tables S9 and S10). There were also no gender differences either within or between groups. 

### 3.8. Percentage of Energy from Carbohydrate, Fat and Protein PKU vs. Control (Includes Energy Intake from Food)

From 6 months of age the mean percentage of energy from protein was consistent at 14–15% for PKU, whilst in control children it gradually increased from 8% (at 4 months) to 17% (at 24 months). Carbohydrate as a percentage of energy steadily increased in the PKU group from 49% (at 4 months) to 60% (at 24 months) whilst the control group had a more variable and lower intake of energy (47–54%) supplied by carbohydrate. Similarly, fat as a percentage of energy was 34–38% for the control group from 6 months of age but in the PKU group energy from fat declined from 40% (at 4 months) to 25% (at 24 months) in line with the increase in energy from carbohydrate.

## 4. Discussion

Children with PKU had comparable growth patterns and similar energy intakes to control children during their early years with all growth parameters within normal range for age. There was a non-significant trend for boys with PKU to grow at a slower rate than control boys and girls with PKU as has previously been seen [4], although still within normal growth parameters. In addition, children with PKU exhibited higher linear growth compared with controls. It has previously been reported that girls with PKU initially show accelerated growth up to 8 years of age followed by slower height progression and a slightly lower final height than the general population [4,15]. In addition, there is some evidence in PKU that girls are more prone to overweight and obesity in the teenage and adult years than boys [26,27,28,29], although it is unclear whether this is associated with early nutrition. Whilst girls with PKU did appear to be taller and had a higher weight and BMI than boys in this study, results did not reach significance possibly due to small subject numbers. 

Children with PKU were weaned on average one month earlier than control children. Whilst some studies have linked early weaning to rate of weight gain, a recent systematic review found that when adjusted for type of milk/formula consumed, there was no significant difference [26]. Certainly there was no evidence that the children with PKU, who were weaned earlier than controls in this study, gained weight (or BMI) any faster. 

Energy and protein intake from protein substitutes for the PKU group remained relatively constant at each age. By 2 years of age, weaning protein substitute contributed approximately 75% of protein intake and 20% of energy intake whilst natural protein from food contributed a mean of 20% of protein intake and 75% of energy intake, with Phe-free infant formula making up the deficit. Timely introduction of appropriate low Phe weaning foods was important to ensure any energy deficit was met. 

In the 1990s and early 2000s despite improvements in dietary treatment and improved protein intake, studies from the USA, Germany, France, the Netherlands, Spain and Australia reported impaired linear growth in PKU [4,5,7,27,28,29,30,31]. One study identified adequate weight gain but moderate growth retardation in infants with PKU in the first 2 years of life compared with the normal population. This was more pronounced in boys but catch-up occurred later [4]. They suggested it might be due to vitamin or trace element intake or adaptation to supplementary feeding during weaning. More recently, a retrospective Spanish study identified inadequate weight and height from 0–2 years of age [10] and a retrospective German study also reported divergent growth in PKU although they acknowledged that in the last 20 years this problem had lessened [11]. It is difficult to compare results of early studies with current data as the protein source (casein hydrolysate vs amino acid) and dose differed. 

In contrast, our prospective study demonstrated that subjects with PKU had higher linear growth z-scores than controls, possibly more so in girls. This gender difference is not uncommon in humans and in other animals. There is evidence to suggest that girls are less likely to deviate from their growth curves under adverse nutritional or environmental conditions than boys [4]. Why infants with PKU might grow faster than control children is unclear, although our study suggests that protein intakes in these children with PKU were adequate. Parental heights were not a confounding factor as these were similar across both groups. It may be that different international therapeutic approaches to treatment in PKU may account for some of the differences in growth identified in earlier studies, as well as improvements in the micronutrient content of protein substitutes [12]. 

There have been concerns in PKU about the absorption and utilisation of artificial sources of protein compared with natural protein and the effect this might have on growth [8,32]. Some studies have suggested that there is no correlation between Phe intake, blood Phe levels and growth deficits in PKU [4,5,7] whilst other studies have eliminated protein or energy malnutrition and endocrine causes [7,8,10]. One study demonstrated that natural protein intake rather than total protein intake was an important factor in head circumference growth in PKU but did not influence linear growth [8]. Certainly, natural protein intakes in our study group at a mean of 0.7–0.8 g/kg (20–23% of protein intake), exceeded those in other prospective studies, which demonstrated normal growth [17]. 

In PKU, as lean body mass is correlated with natural protein intake it is important to maximize natural protein intake to tolerance, although this is sometimes challenging in toddlers [13,17] with or without a metabolic condition. Similarly, an apparent ‘safe’ total protein to energy ratio of 3.0–4.5 g/100 kcal has been reported as contributing to a more favourable body composition [13], and this is consistent with our children with PKU who had a ratio of 3.4–3.7 g/100 kcal. 

Total energy intake as a percentage of DRVs was in fact remarkably similar at all ages in both study groups although, from 12 months of age the balance of carbohydrate and fat as a percentage of energy deviated, with the PKU group consuming a higher carbohydrate intake and the control group a higher fat intake. An increased consumption of carbohydrate compared with children without PKU has previously been reported associated with the high intake of energy-dense, nutrient-poor Phe-free special foods (necessary to meet the energy requirements for metabolic stability) [33,34,35]; although previously it was unclear at what time this deviation might occur. There are concerns that this may impact on body composition compared with regular diets [32]; although data around this topic in non-PKU infants are currently too limited to draw conclusions [36]. 

There were some study limitations. Due to the rarity of PKU and the random nature of any new diagnoses during the study period, gender groups were not equally divided such that some gender differences, whilst not statistically significant, were based on smaller subject numbers, particularly at 4 months of age, thereby making statistical comparisons unreliable. In addition, self-reported dietary data are renowned for under-reporting food intake, although the errors associated with this were the same for both groups. As dietary intake data was based on one 24 h record for each child at each age, it may not represent the norm for that child, although caregivers were advised to record food intakes on days without any illness or abnormal food intake. This method of dietary intake collection also enabled a high rate of return. For the PKU group, food intake generally matched the daily prescribed intake for protein substitute and natural protein (50 mg Phe exchanges); this was probably associated with the intensity of dietary counselling and interventions, the ability to control intake in children and highly motivated caregivers [17,37]. Differences in data collection and reporting were minimised by having the same dietitian follow up most of the subjects in each group. Much of the data on growth in PKU in large cohorts has been retrospective data spanning many years. Whilst this study included fewer subjects, data was collected systematically and prospectively. 

This group of children with PKU, who were weaned onto a second-stage semi-solid protein substitute, demonstrated normal growth compared with their non-PKU peers. It is probable that developments in dietary treatment have led to significant improvements in early growth in PKU. Further work looking at longitudinal growth, body composition, nutrient intake and dietary patterns of children with PKU in early childhood and adolescence would be beneficial in establishing if trends in growth, energy and protein intake continue to follow that of control children and what effect, if any, this may have on their long-term health outcomes. 

## Figures and Tables

**Table 1:** Total energy intake (kcal/day) and percentage of total energy intake attributed to protein substitutes (infant and weaning) and food (mean (range)).

Age Number Subjects	% from Weaning Protein Substitute (WPS)	% from Phe-Free Infant Formula	% from Food	Total kcal/day
4 months *n* = 10	3 (2–4)	49 (31–62)	48 (35–65)	653 (395–831)
5 months *n* = 20	6 (2–11)	52 (30–74)	43 (18–67)	709 (482–1082)
6 months *n* = 19	9 (3–19)	55 (25–75)	37 (16–60)	716 (529–1020)
7 months *n* = 20	11 (5–19)	49 (0–69)	40 (17–85)	802 (583–1187)
8 months *n* = 20	13 (5–22)	44 (0–68)	44 (21–86)	864 (643–1375)
9 months *n* = 19	14 (7–25)	39 (0–69)	47 (21–82)	939 (592–1320)
10 months *n* = 18	16 (7–24)	30 (0–61)	55 (26–84)	983 (610–1558)
11 months *n* = 17	17 (10–26)	29 (0–50)	54 (27–84)	970 (703–1478)
12 months *n* = 19	18 (7–29)	23 (0–49)	59 (35–83)	978 (621–1420)
15 months *n* = 19	18 (14–28)	17 (0–55)	65 (27–85)	1160 (768–1509)
18 months *n* = 20	21 (14–40)	11 (0–44)	67 (42–85)	1155 (595–1511)
24 months *n* = 19	22 (8–38)	5 (0–32)	73 (55–84)	1320 (682–1910)

## Supplementary Materials

The following are available online at https://www.mdpi.com/2072-6643/11/3/552/s1, Table S1: Longitudinal modelling results for Weight z-scores; Table S2: Longitudinal modelling results for Head Circumference z-scores; Table S3: Longitudinal modelling results for BMI z-scores; Table S4: Longitudinal modelling results for Length z-scores; Table S5: Longitudinal modelling results for Length-for-age-difference; Table S6: Longitudinal modelling results for total Protein Equivalent g/kg/day; Table S7: Longitudinal modelling results for Protein Intake g/100 kcal; Table S8: Longitudinal modelling results for Carbohydrates g/kg/day; Table S9: Longitudinal modelling results for Total Energy kcal/day; Table S10: Total energy intake as a percentage of DRV(25) for PKU and control groups and males vs females (mean (range)).
