# Supplementary material for: Growth, Protein and Energy Intake in Children with PKU Taking a Weaning Protein Substitute in the First Two Years of Life: A Case-Control Study
Source: Nutrients. 2019 Mar 5;11(3):552. doi: 10.3390/nu11030552 (PMC6471165; doi:10.3390/nu11030552)
Supplement: Supplementary file 1 [file nutrients-11-00552-s001.pdf]

Supplementary

Details of the mixed models used to model the change in anthropological measures over time. The trend over time is analysed to allow for a linear relationship over time with a single term for time. Longitudinal mixed modelling is applied including a random intercept and slope terms.

Table 1. Longitudinal modelling results for Weight z-scores.

	est	Std. dev	t-value	*p*-value
(Intercept)	0.135	0.223	0.608	0.652
Time	0.042	0.015	2.893	0.032
Gender (Male vs Female)	–0.204	0.191	–1.069	0.472
Treatment (PKU vs Control)	–0.101	0.175	–0.574	0.664
Time: Gender Interaction	–0.020	0.015	–1.320	0.375
Time: PKU Interaction	0.004	0.014	0.314	0.752

Table 2. Longitudinal modelling results for Head Circumference z-scores.

	est	Std. dev	t-value	*p*-value
(Intercept)	–0.405	0.203	–1.990	0.163
Time	0.311	0.192	1.614	0.271
Gender (Male vs Female)	–0.173	0.181	–0.952	0.519
Treatment (PKU vs Control)	0.009	0.014	0.643	0.639
Time:Gender Interaction	–0.039	0.012	–3.218	0.016
Time:PKU Interaction	–0.001	0.015	–0.047	0.827

Table 3. Longitudinal modelling results for BMI z-scores.

	est	Std. dev	t-value	*p*-value
(Intercept)	–0.041	0.243	–0.170	0.795
Time	–0.077	0.206	–0.372	0.734
Gender (Male vs Female)	–0.675	0.188	–3.591	0.006
Treatment (PKU vs Control)	0.036	0.015	2.405	0.083
Time:Gender Interaction	0.025	0.014	1.830	0.205
Time:PKU Interaction	0.019	0.015	1.232	0.409

Table 4. Longitudinal modelling results for Length z-scores.

	est	Std. dev	t-value	*p*-value
(Intercept)	0.316	0.214	1.475	0.319
Time	0.019	0.015	1.278	0.391
Gender (Male vs Female)	–0.348	0.200	–1.739	0.232
Treatment (PKU vs Control)	0.733	0.189	3.872	0.003
Time:Gender Interaction	–0.012	0.016	–0.717	0.611
Time:PKU Interaction	–0.018	0.015	–1.165	0.435

Table 5. Longitudinal modelling results for Length-for-age-difference.

	est	Std. dev	t-value	*p*-value
(Intercept)	1.401	0.454	3.088	0.021
Time	0.022	0.049	0.439	0.711
Gender (Male vs Female)	–0.379	0.508	–0.747	0.599
Treatment (PKU vs Control)	1.488	0.486	3.060	0.022
Time:Gender Interaction	–0.084	0.042	–1.995	0.162
Time:PKU Interaction	–0.029	0.039	–0.752	0.597

Table 6. Longitudinal modelling results for total Protein Equivalent g/kg/day.

	est	Std. dev	t-value	*p*-value
(Intercept)	2.410	0.183	13.160	0.000
Time	0.070	0.013	5.426	0.000
Gender (Male vs Female)	0.065	0.177	0.369	0.735
Treatment (PKU vs Control)	0.543	0.162	3.357	0.011
Time:Gender Interaction	–0.001	0.014	–0.077	0.820
Time:PKU Interaction	–0.042	0.013	–3.230	0.015

Table 7. Longitudinal modelling results for Protein Intake g/100kcal.

	**est**	**Std. dev**	**t-value**	***p*-value**
(Intercept)	2.320	0.170	13.681	0.000
Time	0.102	0.013	7.615	0.000
Gender (Male vs Female)	0.040	0.176	0.225	0.779
Treatment (PKU vs Control)	0.736	0.161	4.566	0.000
Time:Gender Interaction	–0.018	0.014	–1.248	0.403
Time:PKU Interaction	–0.057	0.013	–4.411	0.001

Table 8. Longitudinal modelling results for Carbohydrates g/kg/day.

	**est**	**Std. dev**	**t-value**	***p*-value**
(Intercept)	12.340	0.700	17.619	0.000
Time	–0.074	0.053	–1.397	0.347
Gender (Male vs Female)	1.137	0.699	1.627	0.267
Treatment (PKU vs Control)	–1.490	0.639	–2.331	0.095
Time:Gender Interaction	–0.001	0.056	–0.023	0.833
Time:PKU Interaction	0.232	0.051	4.521	0.000

Table 9. Longitudinal modelling results for Total Energy kcal/day.

	**est**	**Std. dev**	**t-value**	***p*-value**
(Intercept)	644.427	44.814	14.380	0.000
Time	21.690	3.525	6.154	0.000
Gender (Male vs Female)	36.694	44.372	0.827	0.568
Treatment (PKU vs Control)	–72.867	40.121	–1.816	0.209
Time:Gender Interaction	5.233	3.554	1.473	0.319
Time:PKU Interaction	7.811	3.219	2.427	0.080

Table 10. Total energy intake as a percentage of DRV [25] for PKU and control groups and males vs. females (mean (range)).

	**All**		**Males**		**Females**	
	**PKU**	**Control**	**PKU**	**Control**	**PKU**	**Control**
**4m**	118 (72–153)	135 (109–166)	127 (105–153)	124 (109–153)	101 (72–120)	145 (124–166)
**5m**	121 (82–174)	117 (94–173)	126 (82–174)	114 (94–151)	110 (84–122)	119 (100–173)
**6m**	118 (92–164)	122 (96–160)	120 (95–164)	117 (96–148)	114 (92–128)	129 (112–160)
**7m**	116 (81–166)	120 (89–170)	116 (81–166)	115 (89–161)	115 (100–135)	127 (104–170)
**8m**	124 (91–192)	125 (88–168)	125 (91–192)	123 (88–156)	122 (100–168)	129 (94–168)
**9m**	136 (83–184)	137 (96–195)	134 (83–184)	136 (96–195)	140 (104–177)	140 (125–165)
**10m**	142 (85–218)	137 (98–165)	145 (85–218)	133 (98–165)	135 (111–173)	144 (121–165)
**11m**	140 (100–206)	150 (104–186)	140 (100–206)	151 (104–184)	140 (109–184)	149 (123–186)
**12m**	131 (81–186)	133 (91–179)	127 (81–186)	127 (91–167)	139 (110–172)	141 (114–179)
**15m**	155 (107–198)	145 (108–196)	159 (120–197)	141 (108–196)	146 (107–198)	150 (136–162)
**18m**	154 (83–198)	147 (111–208)	164 (123–198)	147 (111–208)	130 (83–181)	147 (115–183)
**24m**	134 (73–194)	123 (79–180)	136 (81–190)	122 (79–180)	131 (73–194)	126 (103–163)
